# Broad-spectrum pH functional chitosan–phosphatase beads for the generation of plant-available phosphorus: utilizing the insoluble P pool

**DOI:** 10.3389/fchem.2024.1359191

**Published:** 2024-04-03

**Authors:** Kasturika Konwar, Himanku Boruah, Rimjim Gogoi, Anudhriti Boruah, Arup Borgohain, Madhusmita Baruah, Subham Protim Gogoi, Tanmoy Karak, Jiban Saikia

**Affiliations:** ^1^ Department of Chemistry, Dibrugarh University, Dibrugarh, Assam, India; ^2^ Department of Soil Science, School of Agricultural Sciences, Nagaland University, Medziphema Campus, Medziphema, Nagaland, India

**Keywords:** chitosan, enzyme, immobilization, phosphorus, fertilizer, acid phosphatase, alkaline phosphatase, sustainable agriculture

## Abstract

Utilization of organic phosphates and insoluble phosphates for the gradual generation of plant-available phosphorus (P) is the only sustainable solution for P fertilization. Enzymatic conversions are one of the best sustainable routes for releasing P to soil. Phosphatase enzyme aids in solubilizing organic and insoluble phosphates to plant-available P. We herein report the preparation of highly functional chitosan beads co-immobilized with acid phosphatase and alkaline phosphatase enzymes via a glutaraldehyde linkage. The dual enzyme co-immobilized chitosan beads were characterized using Fourier-transform infrared (FTIR), thermogravimetric (TGA), and scanning electron microscopy–energy dispersive x-ray (SEM-EDX) analyses to confirm the immobilization. The co-immobilized system was found to be active for a broader pH range of ∼4–10 than the individually bound enzymes and mixed soluble enzymes. The bound matrix exhibited pH optima at 6 and 9, respectively, for acid and alkaline phosphatase and a temperature optimum at 50°C. The phosphate-solubilizing abilities of the chitosan-enzyme derivatives were examined using insoluble tri-calcium phosphate (TCP) for wide pH conditions of 5.5, 7, and 8.5 up to 25 days. The liberation of phosphate was highest (27.20 mg/mL) at pH 5.5 after the defined period. The residual soil phosphatase activity was also monitored after 7 days of incubation with CBE for three different soils of pH ∼5.5, 7, and 8.5. The residual phosphatase activity increased for all the soils after applying the CBE. The germination index of the *Oryza sativa* (rice) plant was studied using different pH buffer media upon the application of the CBE in the presence of tri-calcium phosphate as a phosphate source. Overall, the dual-enzyme co-immobilized chitosan beads were highly effective over a wide pH range for generating plant-available phosphates from insoluble phosphates. The chitosan-enzyme derivative holds the potential to be used for sustainable phosphorus fertilization with different insoluble and organic phosphorus sources.

## Graphical Abstract

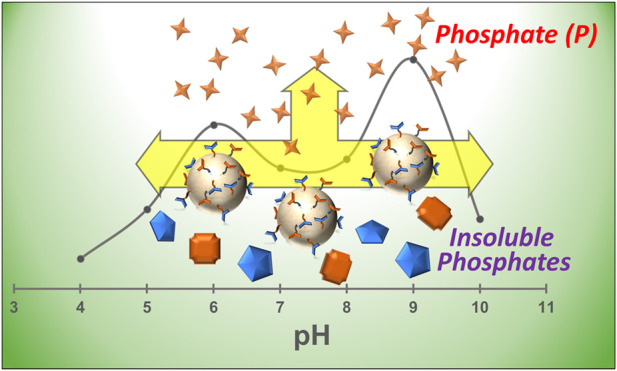

## 1 Introduction

Phosphorus (P) is one of the most essential mineral macronutrients required for healthy plant growth and sustained metabolism. About 80% of the soluble phosphorus fertilizer applied is not available for plant uptake, owing to the tendency of P to form sparingly soluble mineral complexes with different metal ions (Fe^**3+**^, Ca^**2+**^, and Mg^**2+**^) present in soil (Ram et al., 2019; Guera and da Fonseca, 2022). Most of the unused portion runs off to nearby water streams, causing eutrophication (Savic et al., 2022) and toxic algal blooms (Ibarra-Galeana et al., 2017). The presence of low levels of available fractions of this limiting nutrient has threatened productive soil fertility and crop farming worldwide (Sarr et al., 2020). When exposed to P deficiency, plants adapt several mechanisms to increase P acquisition from the complexed P in soil (Vance et al., 2003). However, solubilization of insoluble mineral P complexes and mineralization of insoluble organic phosphates by phosphate-solubilizing microorganisms (PSMs) emerge as the only long-term sustainable approach to increase plant-available P. These **PSMs** function by secreting mineral-dissolving agents, including low-molecular weight organic acids, siderophores, CO_2_, hydrogen ions, and hydroxyl ions, and liberation of enzymes that play a critical role in P cycling (Rodríguez and Fraga, 1999). Enzymes that can dephosphorylate a wide range of organic phosphoesters are termed non-specific acid phosphatases (NSAPs) (Alori et al., 2017). Phosphomonoesterases or phosphatases are the most studied of the NSAPs. Phosphatases can be of two types: acid phosphatases and alkaline phosphatases. Acid phosphatases (ACPs) catalyze the hydrolysis of a broad range of phosphate esters, exhibiting optimal activity in the range of pH 4.00–5.5 (Deiss et al., n. d.). Alkaline phosphatases (ALPs) aid in the solubilization of phosphate groups from variable **insoluble sources** in a pH range of 8.5–9.5 (Jafary et al., 2016). However, applying commercial enzymes in soluble form is not convenient due to problems arising with their recovery and reusability, along with lower stability and inhibited activity in harsh pH and temperature conditions (Sheldon and van Pelt, 2013). Immobilization of enzymes on suitable insoluble solid supports can help mitigate the problems associated with the application of soluble enzymes. Enzyme immobilization, which can be achieved by fixing an enzyme on or within a solid matrix through encapsulation, adsorption, cross-lining, or covalent bonding, is advantageous in providing better stability, and enhanced specificity and activity in harsh environments, followed by better recovery and reusability.

Immobilizing two or more enzymes together on a common support to develop a multifunctional biocatalyst promises to be even more efficient and advantageous through co-immobilization. This can be achieved either by random confinement of two or more enzymes on the same surface on the solid matrix (random co-immobilization) or through covalent bonding between the participants (covalent co-immobilization) (Rios et al., 2016; Moreira et al., 2022). Developing a multienzyme system by co-immobilizing phosphatase enzymes of different specificity in a single support matrix would be very advantageous in providing plant-available P through solubilization and promoting overall plant growth. The system would be expected to work over a wide range of pH and temperature conditions, with better stability and activity.

The various supports used for enzyme immobilization reported in previous works include chitin-like biopolymers (Skwarczyńska et al., 2016), chitosan (Kumari and Rath, 2014; Hua et al., 2023), ceramics, metals, and metal oxides, gelatine, *etc.* (Tihan et al., 2018). Among these, chitosan is the most common support matrix for enzyme immobilization. Chitosan [poly-β(1 4)-2-amino-2-deoxy-D-glucose] is an amino polysaccharide derived from the deacetylation of chitin, the second most abundant natural polymer (Liu et al., 2019; Abidin et al., 2020; Saeed et al., 2020). Its distinctive characteristics, such as non-toxicity, biocompatibility, cost-effectiveness, bio-activity, and robustness in enhancing the enzyme resistance toward chemical degradation, make it an appropriate choice for enzyme immobilization for diverse applications. Chitosan is soluble in organic acid, allowing for the fabrication of gel, film, or beads, making it a suitable support for enzyme immobilization (Altun and Cetinus, 2007). The immobilization can proceed either through entrapment of the enzyme inside the chitosan beads or covalent bonding with chitosan films, aided by the amino groups present, or by cross-linking with glutaraldehyde/glyoxal/glutaric dialdehyde through Schiff’s base formation. Glutaraldehyde is commonly used to activate chitosan beads for covalent attachment of enzymes (Belho et al., 2014). Glutaraldehyde can exist in different reactive forms owing to pH, concentration, *etc.* It can react with a broad range of functional groups in aqueous media through several mechanisms (Manzo et al., 2018).

The aim of this study was to prepare/develop a bifunctional system comprising two enzymes, acid phosphatase and alkaline phosphatase, randomly co-immobilized on prepared chitosan beads pre-activated with glutaraldehyde. The prepared chitosan beads (CBs), glutaraldehyde-activated beads (CBGs), and dual-enzyme co-immobilized chitosan beads (CBEs) were characterized by Fourier-transform infrared (FTIR), thermogravimetric (TGA), and scanning electron microscopy–energy dispersive x-ray (SEM-EDX) analyses. The activity of the bound mixed enzyme was assessed at different pH and temperature conditions. The mixed enzyme co-immobilized on the support matrix was utilized for phosphate solubilization from insoluble mineral P sourced from tri-calcium phosphate (TCP) in three different pH conditions of 5.5, 7, and 8.5 for 25 days to check the effectiveness of the CBE in a broader pH range. The soil residual phosphatase activity was also monitored after 7 days of incubation with CBE. To observe the effect of dual-enzyme immobilized beads on overall plant growth, the growth parameters of the rice plant (*Oryza sativa*) upon application of enzyme co-immobilized beads in different pH conditions were also investigated.

## 2 Materials and methods

### 2.1 Reagents

Chitosan flakes (CAS: 9012-76-4) and hydroxylamine hydrochloride (CAS: 5470−11−1) were purchased from Molychem, Mumbai, Maharashtra, India; oxalic acid dihydrate (CAS: 6153-56-6) and sodium hydroxide (CAS: 1310-73-2) were purchased from FINAR, Gujarat, India; phosphatase, acid (specific activity 20 units/mg solid) from wheat germ (CAS: 9001-77-8), and glutaraldehyde (CAS: 111-30-8) were purchased from Tokyo Chemical Industry Co., Ltd., Tokyo, Japan; alkaline phosphatase (specific activity min. 25 DEA units/mg protein) from calf intestine mucosa (CAS: 9001-78-9) and p-nitrophenylphosphate disodium salt hexahydrate (CAS: 33333−18-4) were purchased from Sisco Research Laboratories Pvt. Ltd., Mumbai, Maharashtra, India; and bovine albumin fraction V powder (98%) for microbiology (CAS: 90604-29-8) was purchased from Loba Chemie Pvt. Ltd., Mumbai, Maharashtra, India.

#### 2.1.1 Preparation of chitosan beads

Chitosan flakes (1.5 g) were dissolved in a 5% oxalic acid solution with continuous stirring at 50°C for 24 h. A gel-like mixture was formed, which was then added dropwise, using a syringe pump, into a solution of 1 M NaOH solution taken in a beaker with continuous stirring to obtain spherical-shaped beads (Juang et al., 2002; Monier et al., 2010). The beads were slowly stirred at room temperature for half an hour to get ripened beads. The beads were then filtered with Whatman no. 1 filter paper and thoroughly washed with Milli Q water until a neutral pH was obtained. Finally, the beads were dried and stored at room temperature for further analysis.

#### 2.1.2 Activation of the prepared chitosan beads

Glutaraldehyde was used as a cross-linker for activating the prepared chitosan beads (Gonawan et al., 2022). The dried chitosan beads were incubated with a 4% v/v glutaraldehyde solution at room temperature for 48 h. The activated beads were collected by filtering with Whatman no. 1 filter paper, followed by washing with modified universal buffer (MUB) of pH 7.0 to remove unbound glutaraldehyde from the surface of the beads. The resultant glutaraldehyde-activated beads (CBGs) were dried and stored at 4°C until further use.

#### 2.1.3 Immobilization of enzyme on activated chitosan beads

The CBGs were used as a support matrix to immobilize acid phosphatase from wheat germ and alkaline phosphatase from calf intestine mucosa. The activated beads were incubated in a 0.5 mg/mL solution of acid phosphatase and alkaline phosphatase separately prepared in MUB of pH 7.0 and kept for 36 h at 4°C. For random co-immobilization, the activated beads were incubated in a solution of acid phosphatase and alkaline phosphatase mixed in a 1:10 weight ratio prepared in MUB of pH 7.0 and kept for 36 h at 4°C. The beads were then collected and gently washed two to three times with MUB of pH 7.0 to remove the unbound enzymes from the surface of the beads. The immobilized enzyme beads were stored at 4°C until further use (Cosnier et al., 2006).

### 2.2 Characterization

The surface morphology and EDX spectra of the samples were recorded using a JEOL JSM-IT300 scanning electron microscope. The FTIR analyses of the samples were performed in an FTIR spectrometer (Agilent Model no: Cary 630, United States; SL. no. MY20192018) in the range of 400–4,000 cm^−1^. The TGA thermograms of the samples were obtained using a thermogravimetric analyzer (Leco TGA701, ASTM D7582- 15) in the temperature range of 25°C–700°C with a heating rate of 20°C/min. A Multiskan SkyHigh Microplate Spectrophotometer (A51119700DPC, Thermo Fisher Scientific, Pvt. Ltd., United Kingdom) in the range of 300–600 nm was used for all spectrophotometric assays.

#### 2.2.1 Determination of the percentage of immobilization

The amount of enzyme (acid phosphatase and alkaline phosphatase) loaded on the activated chitosan beads was determined through a spectrophotometric assay. After removing the beads from the enzyme solution, the amount of enzyme left in the solution was estimated using the Bradford assay for protein estimation using BSA as standard. The percentage immobilization was calculated by the following equation (Srivastava and Anand, 2014):

$$\%\;immobilization=\frac{Total\;activity\;of\;immobilized\;enzyme}{Total\;activity\;of\;soluble\;enzyme}\times 100.$$ (1)

#### 2.2.2 Activity assay for co-immobilized acid phosphatase and alkaline phosphatase chitosan beads

The activity of the co-immobilized enzymes was assayed by adding the required number of CBE beads into a mixture of equal amounts of 5.6 mM pNPP solution as the substrate and MUB at the desired pH. The reaction mixture was incubated at 30°C for 30 min. The beads were then withdrawn, and 0.5 M NaOH solution was added to the reaction mixture to stop the reaction. The absorbance of the liberated p-nitrophenol was recorded at 410 nm (Belho et al., 2014; Srivastava and Anand, 2014).

### 2.3 Effect of pH and temperature on immobilization

A comparative study was conducted to determine the activity of the immobilized enzymes, that is, acid phosphatase, alkaline phosphatase, and mixed enzyme. Various pH values ranging from 4.0 to 10.0 were employed, using MUB to obtain the optimum pH for the enzymes. The activities of the soluble (mixed) and immobilized (CBE) enzymes were compared at different pH (4.0–10.0). In addition, the individual enzyme immobilization on CB (CB-ACP and CB-ALP) was compared with CBE to ascertain the workability of the CBE sample over a wide pH range. The effect of temperature on the activity of both soluble and immobilized enzymes was also studied by incubating the reaction mixture at different temperatures ranging from 10°C to 60°C to obtain the optimum temperature (Cooney, 2011).

### 2.4 Application of immobilized enzyme in solubilization of tri-calcium phosphate

To test for solubilization of phosphate from mineral P sourced from TCP, a 10 mL, 0.05 g/mL solution of TCP was prepared in a buffer solution with pH 5.5, 7, and 8.5. To each pH solution, 1 mL solution of TCP and two beads of each type immobilized with acid phosphatase (CB-ACP), alkaline phosphatase (CB-ALP), and the mixed enzyme (CBE) were added. One control solution in each pH was taken where no beads were introduced. Solution volumes of 0.2 mL were withdrawn from the systems at definite time intervals to test for phosphate released through spectrophotometric assay (He and Honeycutt, 2005).

### 2.5 Soil residual phosphatase activity

The viability and the efficacy of the multi-enzyme-loaded beads (CBE) were tested in soil. Three different soil samples of pH ∼5.5 (Soil sample: A), 7 (Soil sample: B), and 8.5 (Soil sample: C) were collected from different sources. Soils of pH 5.5 and 7 were collected from Dibrugarh University, Assam, India, and nearby areas. Soil of pH 8.5 was generously received from West Bengal, India. The soil acid phosphatase and soil alkaline phosphatase activities were determined using a modified protocol reported by Acosta-Martínez and Tabatabai (2000). During the experiment, 1 g moist soil dispersed in 4 mL of universal buffer of pH 6.5 for acid phosphatase and pH 11 for alkaline phosphatase was mixed with 1 mL pNPP solution. The mixture was shaken gently for some time and then incubated at 37°C for 1 h. After incubation, 1 mL 0.5 M CaCl_2_ and 4 mL 0.5 M NaOH solution were added to stop the reaction. Following that, the solution was filtered with Whatman no.1 filter paper, and the absorbance was recorded at 410 nm (Jin et al., 2016). To monitor the efficacy of the CBE, beads were incubated with the respective soil for 7 days, and the acid and alkaline phosphatase activities were monitored afterward for the relative comparison.

#### 2.5.1 Germination test

A germination test is an effective method of investigating the interaction of plant growth-promoting (PGP) bacteria and seeds (Ibrahim and Ikhajiagbe, 2021). The seeds of the rice plant collected were first surface sterilized with 6%–7% NaClO solution for 20 min and then rinsed three times with sterile distilled water. To examine seed germination, ∼100–200 seeds were randomly placed in a Petri dish and then covered with circular pieces of filter paper. This was followed by the addition of 3 mL TCP solution in MUB (5 mg/mL), with six beads each for three different pH 5.5, 7, and 8.5. For each pH, one sample without the addition of beads was taken as a control. The Petri dishes were then placed in a plant growth chamber, keeping the humidity level at 60% and the temperature at 30°C. When the germ length approached 1 mm length, the seed was considered germinated. The seed germination was observed by examining the time of appearance of the primary leaf (coleoptile) every 6 h. The % germination was calculated from Equation 3.

The germination index (GI) was calculated by the method of Wang et al. (2010) as follows:

$$GI=\sum G_{t}/t,$$ (2)

where $G_{t}$ denotes the number of seeds germinated on day t. Seedlings showing germination per dish were randomly selected after 9 (nine) days of germination (Anupama et al., 2014).

$$Germination\;percentage\;\left(\%\right)=\frac{Number\;of\;germinated\;seeds\times 100}{Total\;number\;of\;seeds\;sowed}.$$ (3)

#### 2.5.2 Biomass parameters

##### 2.5.2.1 Seedling length (cm)

Seedlings showing germination from the studied system were selected to measure the total seedling length on day 9. The length of the seedling was measured from the collar region to the tip of the primary leaf. The mean seedling length was expressed in centimeters (Anupama et al., 2014).

##### 2.5.2.2 Seedling vigor index (VI)

To estimate the vigor, shoot, and root length of individual seedlings, the vigor index (VI), expressed in whole numbers, was determined from Equation 4 (Morsy, 2021):

$$Vigor\;Index\;\left(VI\right)=Germination\;\left(\%\right)\times Seedling\;length\;\left(cm\right).$$ (4)

### 2.6 Statistical analysis

The one-way analysis of variance (ANOVA), followed by the least significant difference (LSD) test, was used to test for significant differences between the mean responses of the control group and treatments (CB and CBE) used in investigating the growth in seedling length of rice seeds (*O. sativa*) in three different pH values (5.5, 7, and 8.5) at a 95% level of confidence.

## 3 Results and discussion

### 3.1 Immobilization of acid phosphatase and alkaline phosphatase on glutaraldehyde-activated chitosan beads

**FIGURE 1:**
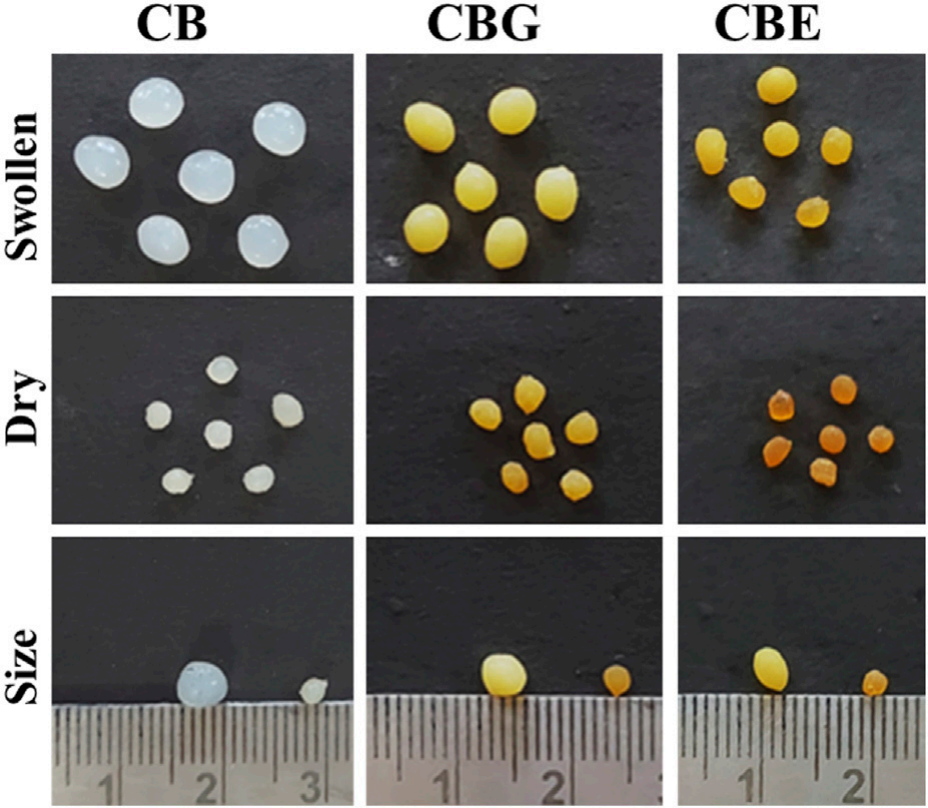
Optical images for chitosan beads: normal CBs, glutaraldehyde-activated chitosan beads (CBGs), and mixed-enzyme-immobilized chitosan beads (CBEs).

Enzymes are immobilized on support matrices, such as chitosan beads and chitosan nanoparticles, for diverse functions. Co-immobilization with two or three enzymes imparts multifunctionality. In these cases, activating the carrier matrix prior to immobilization provides better operational stability and improved performance. Aldehyde groups are introduced on the surface of chitosan by glutaraldehyde activation. The immobilization process is carried out under neutral pH conditions. As calculated from Equation 1, immobilization (%) values for acid and alkaline phosphatase are 66.2% and 13.26%, giving a total value of 79.5% immobilization of the mixed enzyme on the activated chitosan surface. Figure 1 shows the optical images of the prepared CB, chitosan beads activated with glutaraldehyde (CBG), and mixed enzyme (ACP and ALP) immobilized chitosan beads (CBE) in their dry and swollen states. Upon activation, the color of the chitosan beads turned yellowish brown, which became more intense after immobilization with the enzymes. The color intensification may be attributed to the reaction of primary amine and the aldehydic group through Schiff’s base formation, giving unsaturated bonds C=N, C=O and C=C in the cross-linking structure (Belho et al., 2014; Galan et al., 2021). In the literature, the intensity of the color of the chitosan beads was found to increase with increasing glutaraldehyde concentration (Belho et al., 2014). As is apparent in the figure, the swollen sizes of the activated and enzyme-immobilized beads are smaller than the normal chitosan beads. This can be explained on the basis of alteration in the chitosan structure upon the introduction of glutaraldehyde. This results in the consumption of the amino groups during the cross-linking reaction, reducing the active sites present and, consequently, lowering the capacity of the chitosan beads to bind the solvent molecules. The extent of swelling was observed to decline with increasing glutaraldehyde concentration in the work reported by Galan et al. (2021). Similar reasoning can be attributed to the case of enzyme-immobilized beads, in which the enzymes bind to the active sites, rendering those sites unavailable for solvent binding.

**FIGURE 2:**
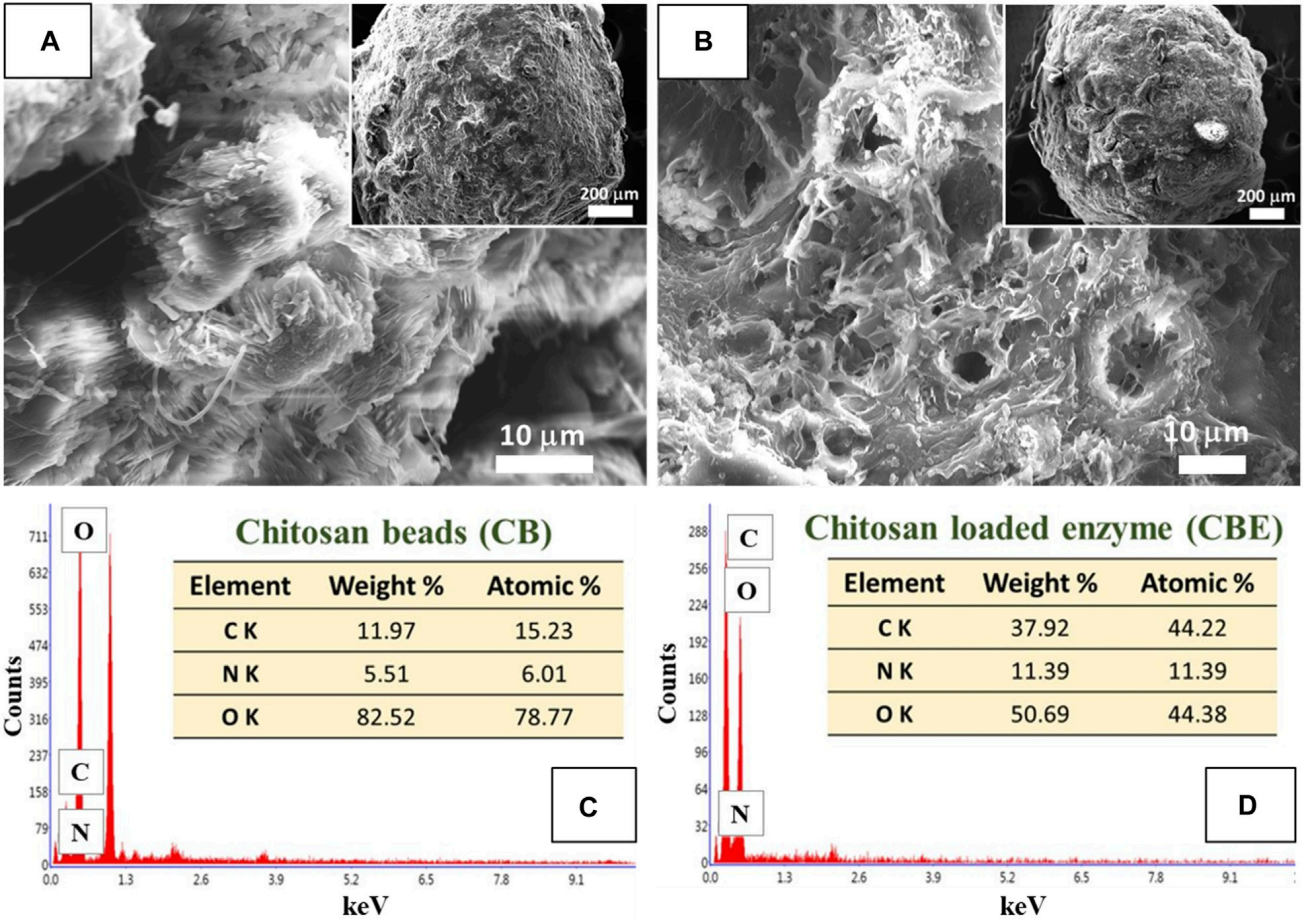
SEM images for **(A)** chitosan beads (CBs) and **(B)** enzyme-immobilized beads (CBEs). EDX spectra for **(C)** CBs and **(D)** CBEs.

The surface morphology of the prepared CBs and CBEs was investigated using SEM analysis. The change in surface properties of the CBs upon immobilization post-activation with glutaraldehyde is observed in the SEM micrographs (Figures 2A,B). A porous structure was observed on the surface of the CBs; similar structures are usually found in this type of polymeric material (Benucci et al., 2019; Moreira et al., 2022). The increase in roughness and porosity of the CBs after immobilization illustrates the chemical modification brought upon by glutaraldehyde and successful enzyme binding on the support matrix (Monier et al., 2010). The chemical compositions of the beads were assessed from the EDX spectra of the samples (Figures 2C,D). The proportions of C and N increased from 15.23% to 6.01% in CBs to 44.22% and 11.39%, respectively, in CBEs, whereas the content of O decreased from 78.77% to 44.38%. This can be attributed to the increase in organic portion upon introduction of glutaraldehyde and enzyme as the surface of the activated chitosan beads is covered with the enzymes, indicating successful immobilization (Moreira et al., 2022).

**FIGURE 3:**
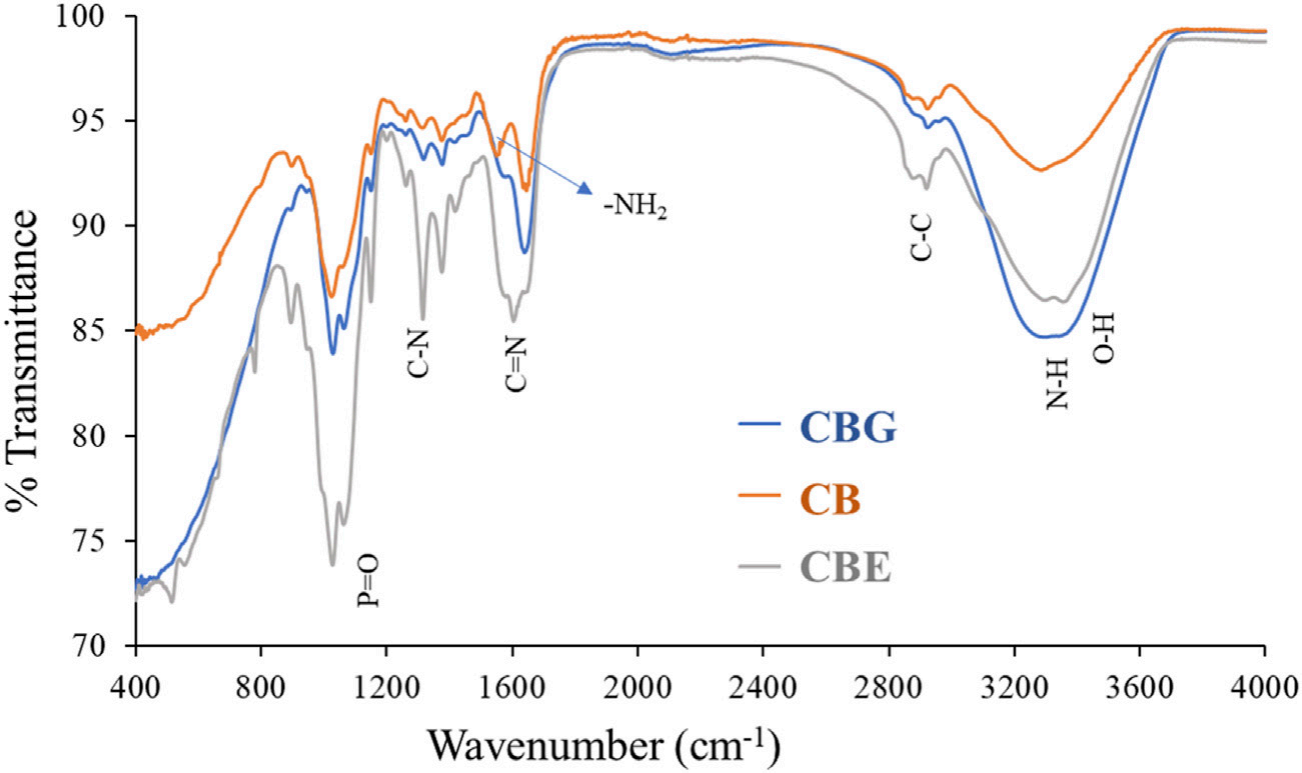
FTIR spectra for CB, CBGs, and CBEs.

FTIR analysis was performed to investigate the nature of interaction existing in the structures of chitosan (CB), glutaraldehyde-activated chitosan (CBG) and dual enzyme-loaded chitosan (CBE) samples. From the FTIR spectra depicted in Figure 3, all three samples exhibit peaks for the stretching vibration of an O-H bond overlapped with N-H bonds of approximately 3,400 cm^−1^. The band around 2,880 cm^−1^ can be assigned to the stretching vibration of a C-C bond in the chitosan polysaccharide structure in all three samples. The gradual disappearance of the characteristic vibration for an -NH_2_ bond in the chitosan structure is observed in the activated and enzyme-loaded samples, signifying efficient cross-linking by glutaraldehyde in both structures. The absorption band at approximately 1633 cm^−1^ in CBs may be assigned to N-H stretching. For the CBGs, the peak at 1663 cm^−1^ might be correlated with a C=N bond, owing to the imine reaction between the chitosan amino groups and the glutaraldehyde molecule. Mixed-enzyme-loaded beads exhibited an intense band at approximately 1621 cm^−1^ due to C=N bond stretching. The shifting of the peak and the increase in the intensity of the band at approximately 1676 cm^−1^ indicates an increase in the intensity of the imine cluster present in their structures, owing to the cross-linking of glutaraldehyde and chitosan moieties (Moreira et al., 2022). Glutaraldehyde furnishes an aldehydic group on the chitosan surface, which further immobilizes the enzyme through the formation of a Schiff’s base with the amino groups (Srivastava and Anand, 2014).

**FIGURE 4:**
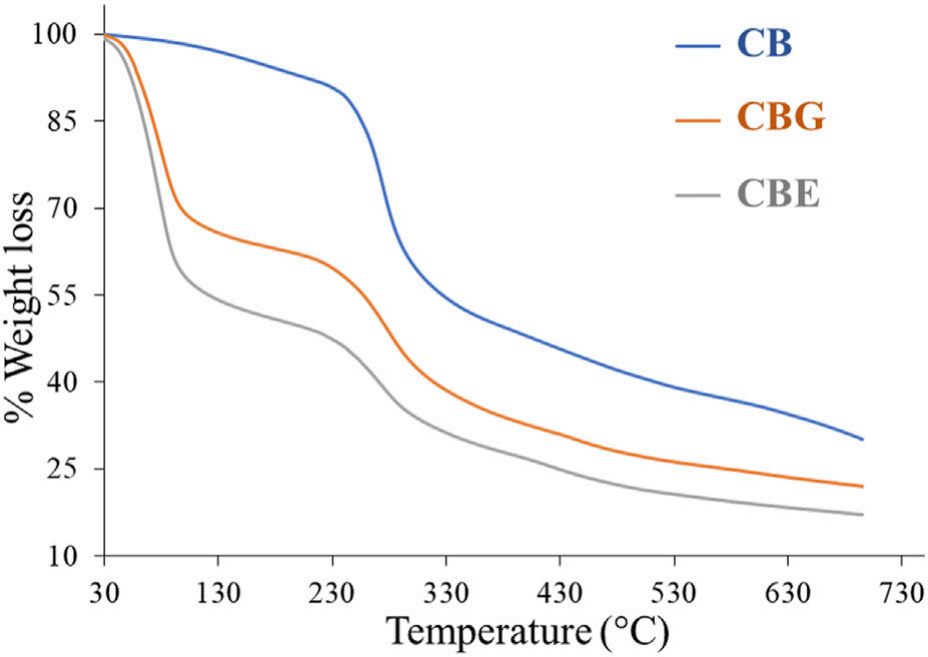
TGA thermogram for CB, CBGs, and CBEs.

The TGA thermogram depicted in Figure 4 demonstrates the weight loss occurring in two steps in all three samples. The first weight loss stage was observed at around 100°C, showcasing a ∼3% weight loss in the CB sample, a ∼30% weight loss in the CBG sample, and a ∼42% weight loss in the CBE sample due to the desorption of water molecules from the polymeric fiber. The second break was observed at approximately 230°C, exhibiting a ∼40% weight loss for only the chitosan sample, a ∼54% weight loss for the CBGs, and a ∼63% weight loss in the case of the enzyme-loaded sample. The weight loss during the second break was observed to be highest in the enzyme-loaded sample, followed by the CBGs and then the CBs, with the weight loss occurring in a wider temperature range in the former samples, indicating more significant material degradation (Moreira et al., 2022). This may be ascribed to the breakage of chemical bonding between chitosan and glutaraldehyde and the immobilized dual enzymes. As can be understood from the weight loss patterns of the glutaraldehyde-activated and enzyme-loaded samples, the thermal stability decreased upon glutaraldehyde cross-linking. The thermal degradation patterns continue in higher temperatures, demonstrating higher mass loss in activated and enzyme-loaded samples and indicating that immobilization of enzymes occurs on the activated chitosan beads.

### 3.2 Effect of pH and temperature on catalytic activity

#### 3.2.1 Optimum pH

**FIGURE 5:**
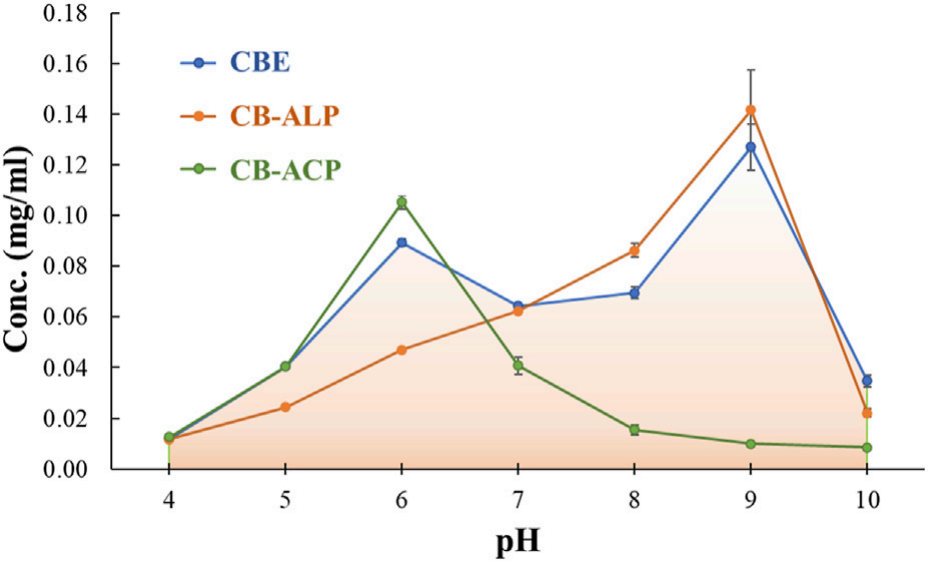
Effect of pH on the relative activity of immobilized acid phosphatase (ACP), alkaline phosphatase (ALP), and mixed enzymes.

The effect of pH on the activity of the bound enzymes was studied by recording the absorbance of the liberated p-nitrophenol from hydrolysis of pNPP in the desired pH ranges. The pH profiles for mixed-bound enzymes (acid phosphatase and alkaline phosphatase) and individually bound acid and alkaline phosphatase are represented in Figure 5. It can be observed that the optimum pH values for acid phosphatase and alkaline phosphatase are 6 and 9, respectively. The dual co-immobilized enzyme system showed optimal activity at both pH 6 and 9 individually assigned for both enzyme components. Although the activity expressed in terms of concentration of liberated p-nitrophenol for mixed enzyme matrix is lower than the individually bound enzyme matrices at the optimal pH values, the activity range of the former is found to be broader, ranging from ∼4−10. The lowering of activity could be assigned to the inhibitory effect introduced by both enzymes for each other. A comparative pH profile for the bound mixed enzyme and the soluble mixed enzyme is depicted in Supplementary Figure S1. Note that the optimal pH did not shift for the bound mixed enzyme when compared to its native form. However, the activity of the immobilized enzyme is much higher than that of the free enzyme, and it also exhibits high activity over a broader pH range. Many literature reports state the shifting of optimal pH values for immobilized enzymes from the soluble form. Acid phosphatase from wheat germ immobilized on CBG showed a shifting of optimal pH from 5 to 5.5 (Belho et al., 2014), whereas acid phosphatase from *Vigna aconitifolia* seeds immobilized on chitosan beads displayed an increase in the optimal pH value from 5 to 7 compared to the soluble form. Beta-galactosidase immobilized on chitosan experienced a shift of 6.3–6.9 to 6.9–7.5 (Carrara and Rubiolo, n. d.). Another report showcased an increase of 1 unit in optimal pH upon immobilization of horseradish peroxidase on modified chitosan beads (Monier et al., 2010). All the shifts witnessed are displaced to more alkaline values. The change in pH is primarily assigned to the modification brought upon by the presence of the support material with variable physical and chemical characteristics in the microenvironment around the enzyme (Altun and Cetinus, 2007). However, several reports also demonstrate no shifting in optimal pH for enzymes post- immobilization. Pepsin immobilized on chitosan beads cross-linked with glyoxal hydrate (Altun and Cetinus, 2007), lipase immobilized on chitosan beads cross-linked with genipin (Chiou et al., 2007), and catalase enzyme immobilized on chitosan beads cross-linked with glyoxal hydrate (Çetinus and Öztop, 2003) showed optimal activity at pH values similar to their soluble form. The presence of cationic support materials tends to shift the pH maximum toward the acidic side, whereas anionic supports tend to shift the pH maximum toward the alkaline side. Chitosan carries no charge in neutral pH. The two bound enzymes, acid and alkaline phosphatase, show high catalytic activity at acidic and alkaline pH values, respectively. In acidic conditions, the amino groups of the chitosan beads might bind hydrogen ions in solution and become positively charged. However, the reduced number of amino groups remaining on cross-linked chitosan beads post-immobilization inherently lowers the number of bound hydrogen ions. This prohibits significant alteration in the enzyme microenvironment and the pH profile in acidic conditions (Altun and Cetinus, 2007). In both cases, the immobilized enzymes exhibited a wider pH range for optimal activity. From Supplementary Figure S1, it is evident that the activity decreases for both soluble and bound enzymes beyond their optimal pH ranges due to structural deformation at harsh pH conditions. This activity loss is more prominent in the case of soluble enzymes. It can be concluded that immobilization on the support matrix provides resistance for the enzymes in harsh pH conditions.

#### 3.2.2 Optimum temperature

**FIGURE 6:**
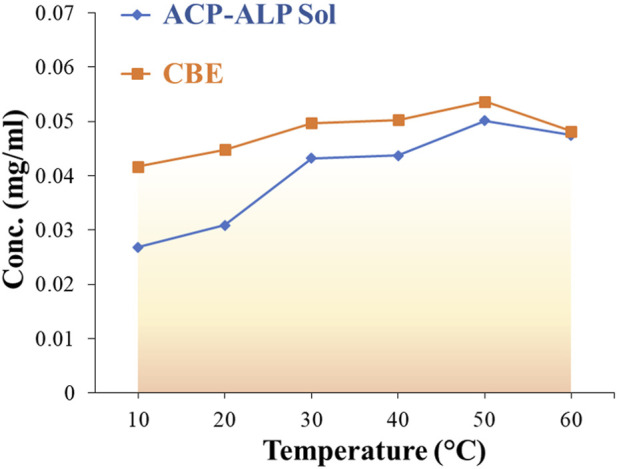
Effect of temperature on the relative activity of free (soluble) and immobilized mixed enzymes.

The effect of temperature on the activity of both soluble and immobilized mixed enzymes was studied over a temperature range of 10°C–60°C. The optimum temperature for both soluble and immobilized enzymes was observed to be 50°C, as depicted in Figure 6. This indicates that immobilization did not alter the temperature maximum for the mixed enzyme. There are reports of shifting of temperature optimum after immobilization on solid supports (Altun and Cetinus, 2007; Belho et al., 2014). In those cases, the immobilization of the enzyme with the support material impacted its conformational flexibility. The support matrix enhances enzyme rigidity, providing stability toward denaturation at high temperatures, thereby shifting the temperature maximum to a higher value. However, immobilization of lipase enzyme on cross-linked chitosan beads reported by Chiou et al. (2007) showcased no alteration in the temperature for optimal activity of the soluble and bound enzymes. The immobilized lipase was observed to exhibit better activities than the free lipase at temperatures lower than 30°C. Above 30°C, the activity of the immobilized lipase declined more than the free enzyme. Similarly, the optimum temperature was observed to rise to higher values in catalase enzyme after immobilization on cross-linked chitosan beads (Akkuş Çetinus and Nursevin Öztop, 2003) and acid phosphatase from *V. aconitifolia* seeds immobilized on chitosan beads (Srivastava and Anand, 2014). In our study, no change in optimum temperature was witnessed post-immobilization, but the bound mixed enzyme showed slightly better activity in a broader temperature range than the soluble counterpart. The increase in structural rigidity and conformational flexibility of the enzymes protects them from unfolding, and immobilization by multipoint attachments to an insoluble matrix enhances the activity of the bound enzymes at the optimum temperature (Jafary et al., 2016). The support material increased the thermal resistance of the enzyme through the formation of covalent bonding between the enzyme and the support and absorption of some fraction of heat by the supporting matrix (Monier et al., 2010; Srivastava and Anand, 2014).

### 3.3 Quantitative estimation of phosphate solubilization

#### 3.3.1 Release of phosphate from tri-calcium phosphate in solution

**FIGURE 7:**
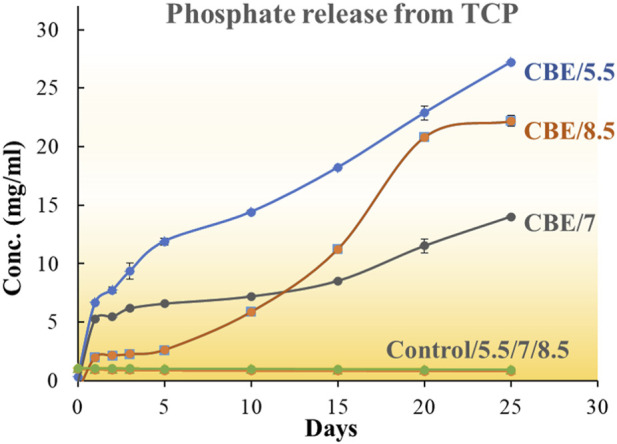
Comparative phosphate release profile for ACP, ALP, mixed enzymes, and control (no enzyme) at pH 5.5.

The effectiveness of the co-immobilized enzyme system in solubilizing tri-calcium phosphate in pH 5.5, 7, and 8.5 was studied. The enzyme-immobilized matrices successfully catalyzed the solubilization of phosphate in all three pH values (Figure 7). In acidic conditions (pH 5.5), the CB-ACPs exhibited optimal activity, liberating 29.36 mg/mL phosphate over 25 days (Supplementary Figure S2). In neutral (pH 7) and alkaline conditions (pH 8.5), the CB-ALPs demonstrated the highest activity, solubilizing 23.774 mg/mL and 27.47 mg/mL phosphate, respectively. The CBEs showed an activity between the CB-ALPs and the CB-ACPs at all three pH values. However, among the three pH values, the CBEs demonstrated the highest activity at pH 5.5, releasing 27.20 mg/mL phosphate during the observed time period.

No existing literature reports the application of enzyme-loaded substrate in insoluble phosphate solubilization. But the role of phosphatase enzymes in mineralization of organic phosphates and solubilization of inorganic phosphates are known. The solubilization of mineral phosphate through the excretion of low molecular organic acids (citric acid, succinic acid, lactic acid, malonic acid, *etc.*) by phosphate-solubilizing bacteria and fungi accompanied by acidification of the media is considered to be the more crucial route. In this regard, the solubilization of calcium phosphate by *Pseudomonas* without the production of organic acid is reported by Illmer and Schinner (1992) and Illmer and Schinner (1995). Some microorganisms reported in the literature function in phosphate solubilization through both organic acid production and secretion of phosphatase enzyme. *Piriformospora indica*, a fungus similar to arbuscular mycorrhiza, is reported to contribute to the solubilization of organic phosphates and polyphosphates through the activity of enzyme acid phosphatase (66 kDa) (Swetha and Padmavathi, 2016). Several studies state that fungi have a higher ability to solubilize mineral phosphates than bacterial species. Fungi synthesize an extensive number of phosphatase enzymes that have characteristic phosphate scavenging ability (Kapri and Tewari, 2010). Phosphatase enzymes have been demonstrated to exhibit higher activity in P-deficient conditions (Häussling and Marschner, 1989). The non-specific acid phosphatases show optimal activity in their ambient pH environments.

The mixed enzyme-loaded chitosan beads in our study have the advantage of working in both acidic and alkaline environments, effectively liberating phosphate from its source in both conditions. However, the liberation of phosphate is observed to be highest at pH 5.5. It might be due to the fact that an acidic environment facilitates the scavenging of phosphates; in that scenario, the acid phosphatase component would be more active and effectively contribute to the liberation of phosphate.

### 3.4 Soil residual phosphatase activity

**FIGURE 8:**
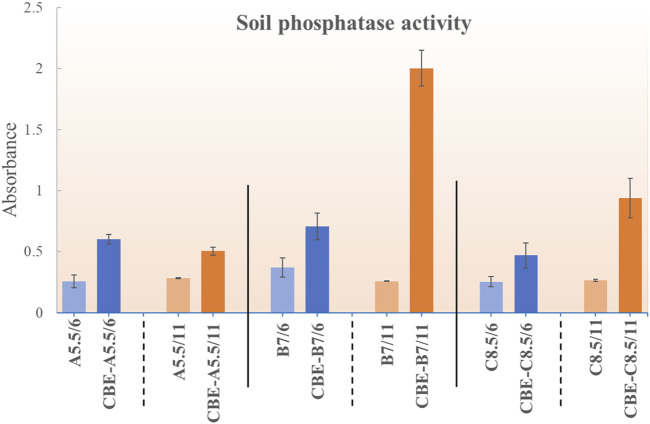
Residual phosphatase activity for CBE in soils of pH 5.5, 7, and 8.5.

The acid and alkaline phosphatase activities of soil were monitored at pH 6 and 11, respectively, for all three soil samples (pH 5.5: A, pH 7: B, and pH 8.5: C). Fresh soil samples were analyzed for phosphatase activity, and respective soils were incubated with enzyme-loaded beads (CBE) for 7 days (Figure 8). For all samples, both the acid and the alkaline phosphatase activity increased. For sample A (soil sample pH 5.5), the relative increment of the acid phosphatase activity (pH 6 buffer) was higher than the alkaline phosphatase activity (pH 11). This could be justified by the fact that for sample A, at a lower pH, the dominant acid phosphatase shows better efficacy, whereas the alkaline pH shows a relatively lower incremental increase. For the B and C samples (at both pH 6 and 11), an incremental increase in activity was observed upon incubation with CBE. The alkaline phosphatase activity was found to increase more than the acid phosphatase for both samples B (pH 7) and C (pH 8.5). From these results, it could be inferred that the prepared CBEs are stable in real soil applications. The activity of CBE was intact even after 7 days of incubation at different soil pH levels. The beads were also stable with no visible change up to 15 days of incubation (see Supplementary Figure S3).

### 3.5 Germination parameters

**FIGURE 9:**
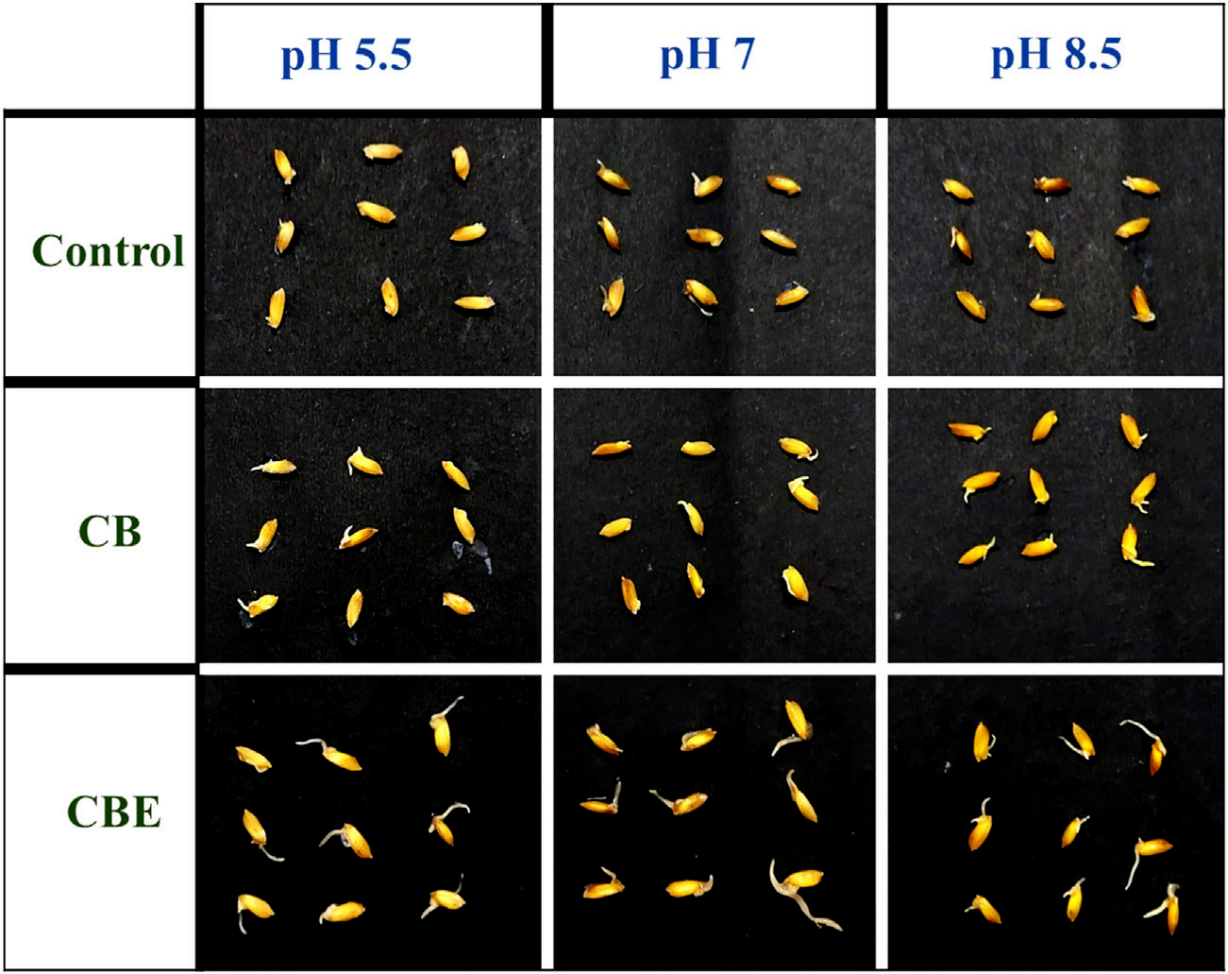
Appearance of the rice (*Oryza sativa*) seed coleoptile after 9 days of treatment with CBs and CBEs.

**FIGURE 10:**
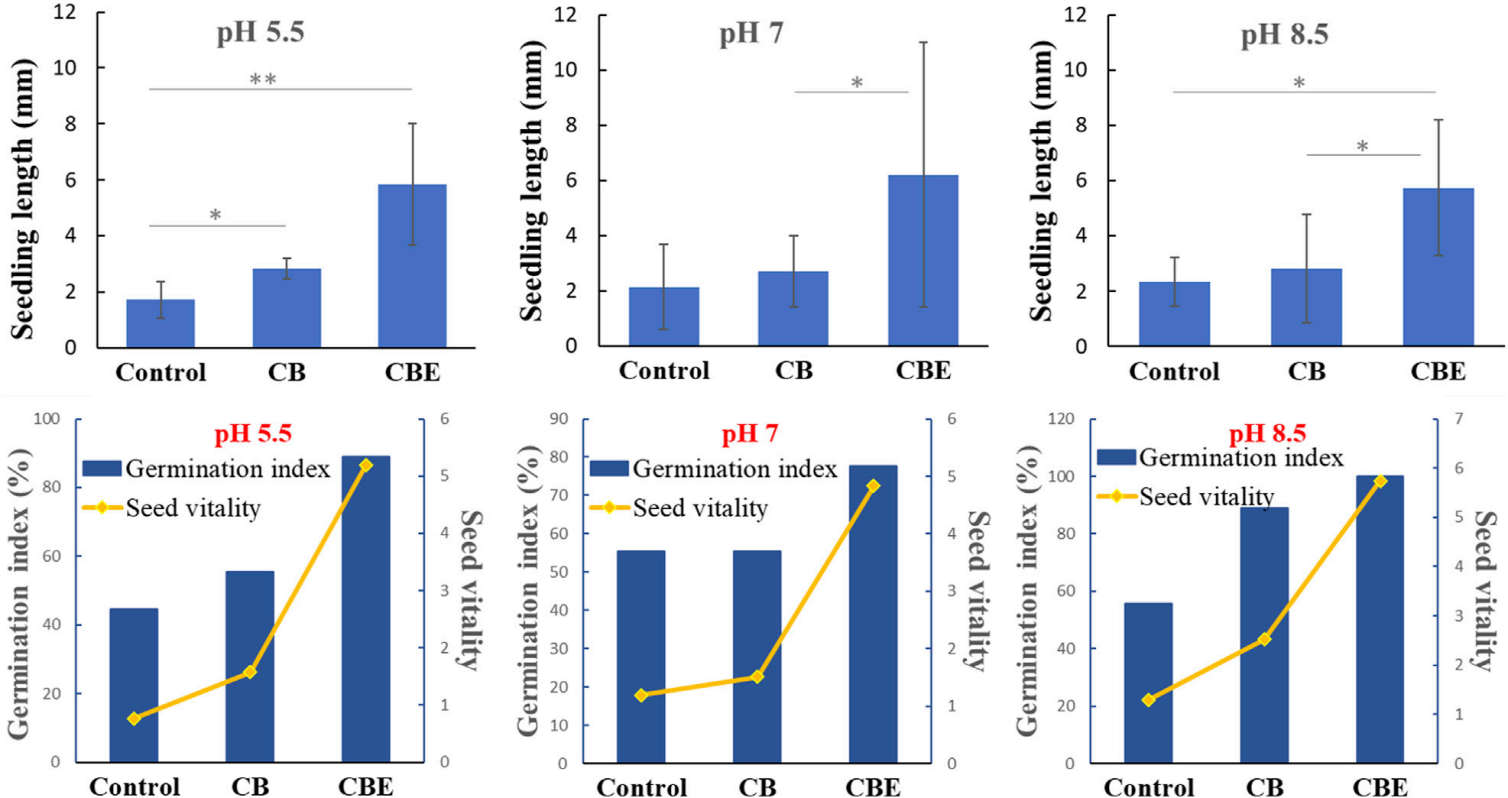
Profile for seedling length and germination index for rice (*Oryza sativa*) seeds after treatment with CBE beads in pH 5.5, 7, and 8.5 (*: significant at the 5% level, **: significant at the 1% level).

The germination of rice seeds post-treatment in three different pH values is depicted in Figure 9. No earlier work has reported on the direct application of enzyme or enzyme-loaded matrix in the investigation of seed germination. Ibrahim and Ikhajiagbe (2021) reported the treatment of rice seeds with three strains of phosphate solubilizing bacteria (PSB) and observed significant growth of the rice seeds. The rice seeds inoculated with *Bacillus cereus* strain showed optimal germination percentage compared to the other two strains, *Proteus mirabilis* and *Klebsiella variicola,* resulting in the accelerated appearance of coleoptile within 24 h of inoculation. In the present study, from the pattern of appearance of coleoptile on the seeds, it is evident that the seeds treated with CBEs exhibit the highest germination rate. The % germination after treatment with CBEs is calculated to be highest at pH 8.5 (Figure 10). The seeds treated with only CBs showed delayed germination, whereas the control samples exhibited almost no germination. This demonstrates the positive impact of the enzyme-loaded beads on the rice seeds. The seedling length is also the longest in the CBE-treated seed samples (Figure 10). Thus, it is apparent that treatment with CBEs significantly enhances the germination parameters of the seed samples. This showcases the ability of the mixed enzyme to be in an active state without undergoing structure denaturation in extreme pH conditions, bring change in the seed microbiome, and improve the germination parameters (Ibrahim and Ikhajiagbe, 2021). The overall germination rate was slower with respect to the time taken for the required change. This may be attributed to the utilization of a buffer solution, which possesses an inhibitory effect on the growth of the coleoptile from the rice seeds, during the germination experiment. Acidic buffers, in general, are reported to inhibit seed germination and cause roots to grow shorter and thicker. All alkaline buffers except CHES-sodium hydroxide are reported to constrain seed germination of *P. contorta, P. glauca*, *etc.* The inhibitory effect of buffer solutions is inherently related to the sensitivity of germination toward the increased osmotic potential of the buffering agents. In extremely acidic conditions, the hydrogen ion concentration is reported to constrict seedling growth (Redmann and Abouguendia, 1979).

### 3.6 Statistical interpretation of seedling length upon treatment with CBs and CBEs

A one-way ANOVA was performed to ascertain the influence of the applied enzyme-loaded beads on the seedling length of rice (*O. sativa*). An LSD test was carried out to test for the significance of the difference between the mean values of control (average seedling length) and the treatments (CB and CBE) used at pH 5.5, 7, and 8.5. The result of ANOVA in the three different pH values is given in Supplementary Table S1.

From the results, *p*-values corresponding to the F-test were found to be 0.00, 0.071, and 0.014, respectively, at pH 5.5, 7, and 8.5, which is significant at the 5% (0.05) level. The presence of a statistically significant difference between the treatment groups is noted.

An LSD test was used to determine the pair-wise comparison of the responses of the groups in the three pH levels, as given in Supplementary Table S2. The table shows that there is a statistically significant difference in seedling length for all the pairs at pH 5.5. However, at pH 7, a significant difference was only exhibited by the pair treated with CBs and CBEs, with a *p*-value of 0.037. At pH 8.5, the CBE and control group pair shows a highly significant difference with a *p*-value of 0.009, whereas the CBE and CB pair shows a significant difference with a *p*-value of 0.011. Thus, the application of enzyme-loaded beads exhibited a discernable effect in the growth of rice seedlings for tests conducted at pH 5.5 and 8.5 compared to the control. This may be attributed to the dominance of the individual enzymes, respectively, in acidic and basic conditions, subsequently presenting optimal results.

## 4 Conclusion

Broad-spectrum pH-active dual-enzyme immobilized chitosan beads were prepared for the effective solubilization of tri-calcium phosphates at different pH levels. The application of CBE beads significantly enhances the germination parameters for *O. sativa* (rice) seeds at pH 5.5, 7, and 8.5. Soil application of CBE beads resulted in enhanced soil residual phosphatase activity for the soils of pH 5.5, 7, and 8.5 up to 7 days post-incubation. These CBE beads hold great potential for solubilizing various insoluble phosphate sources for sustainable generation of plant-available phosphorus.

## Data Availability

The original contributions presented in the study are included in the article/Supplementary Material; further inquiries can be directed to the corresponding author.

## Abbreviations

CB, chitosan bead; CBG, glutaraldehyde-activated chitosan beads; CBE, mixed-enzyme-loaded chitosan beads; FTIR, Fourier-transform infrared spectroscopy; TGA, thermogravimetric analysis; SEM, scanning electron microscope; EDX, energy-dispersive X-ray; TCP, tri-calcium phosphate; ACP, acid phosphatase; ALP, alkaline phosphatase.
