# Supplementary material for: Broad-spectrum pH functional chitosan–phosphatase beads for the generation of plant-available phosphorus: utilizing the insoluble P pool
Source: Front Chem. 2024 Apr 3;12:1359191. doi: 10.3389/fchem.2024.1359191 (PMC11021595; doi:10.3389/fchem.2024.1359191)
Supplement: Supplementary file 1 [file DataSheet1.docx]

Supplementary Material

Broad Spectrum pH Functional Chitosan-phosphatase beads for the generation of plant available phosphorous: utilizing the insoluble P pool

**Kasturika Konwar^1†^, Himanku Boruah^1†^, Rimjim Gogoi^1^, Anudhriti Boruah^1^, Arup Borgohain^1^, Madhusmita Baruah^1^, Subham Protim Gogoi^1^, Tanmoy Karak^2^ and Jiban Saikia^1*^.**

^1^ Department of Chemistry, Dibrugarh University, Dibrugarh - 786004, Assam, India.

^2^ Department of Soil Science, School of Agricultural Sciences, Nagaland University, Medziphema Campus, Medziphema 797106, Nagaland, India.

**Correspondence:** Corresponding Author: jibansaikia@dibru.ac.in

†These authors contributed equally to this work and share first authorship

# Supplementary Figures and Tables

## Supplementary Figures


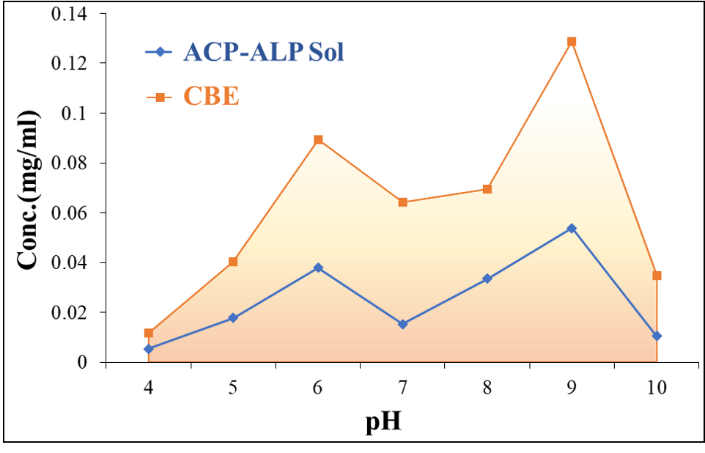


**Supplementary Figure S1.** Effect of pH on the relative activity of free (soluble) and immobilized mixed enzyme (CBE).


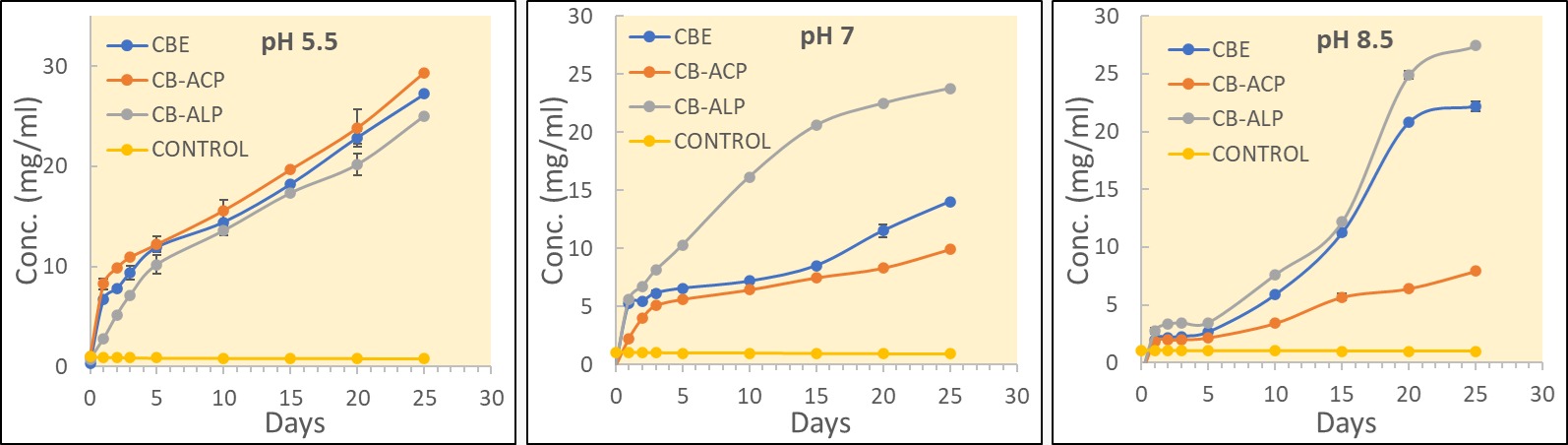


**Supplementary Figure S2.** Comparative phosphate release profile for mixed enzyme loaded chitosan bead (CBE), acid phosphatase loaded chitosan bead (CB-ACP), alkaline phosphatase loaded chitosan bead (CB-ALP) and control (no enzyme) at pH 5.5, 7 and 8.5.


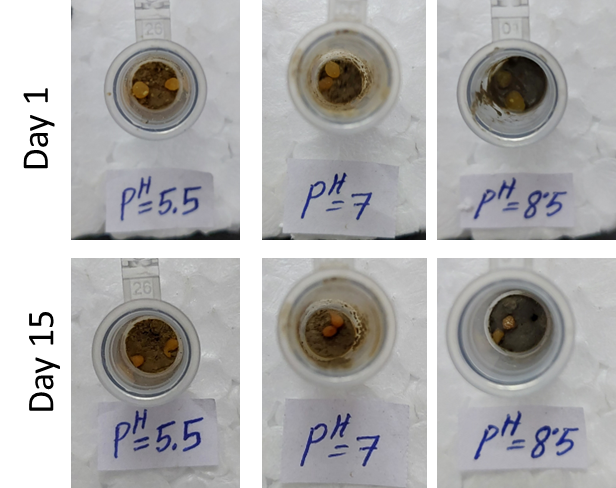


**Supplementary Figure S3.** CBE Beads kept in soil of different pH for 15 days showing the stability of the beads.

## Supplementary Tables

Table S1: One way ANOVA for the responses (seedling length)

|  |  | Sum of Squares | df | Mean Square | F | P-value |
| --- | --- | --- | --- | --- | --- | --- |
|  | Between Groups | 32.068 | 2 | 16.034 | 54.660 | .000 |
| pH – 5.5 | Within Groups | 2.640 | 9 | 0.293 |  |  |
|  | Total | 34.709 | 11 |  |  |  |
|  | Between Groups | 10.516 | 2 | 5.258 | 3.604 | 0.071 |
| pH – 7 | Within Groups | 13.128 | 9 | 1.459 |  |  |
|  | Total | 23.644 | 11 |  |  |  |
|  | Between Groups | 37.858 | 2 | 18.929 | 7.146 | 0.014 |
| pH – 8.5 | Within Groups | 23.842 | 9 | 2.649 |  |  |
|  | Total | 61.699 | 11 |  |  |  |

Table S2: LSD for multiple comparison

|  | Dependent Variable: Seedling length | | | | | |
| --- | --- | --- | --- | --- | --- | --- |
|  | (I) Treatment | | (J) Treatment | Mean Difference (I-J) | Std. Error | P-value |
|  | | Control | CB | -1.095500^*^ | .382978 | .019 |
|  | |  | CBE | -3.883250^**^ | .382978 | .000 |
|  | | CB | Control | 1.095500^*^ | .382978 | .019 |
| pH – 5.5 | |  | CBE | -2.787750^**^ | .382978 | .000 |
|  | | CBE | Control | 3.883250^**^ | .382978 | .000 |
|  | |  | CB | 2.787750^**^ | .382978 | .000 |
|  | | Control | CB | .237250 | .854025 | .787 |
|  | |  | CBE | -1.856500 | .854025 | .058 |
|  | | CB | Control | -.237250 | .854025 | .787 |
| pH – 7 | |  | CBE | -2.093750^*^ | .854025 | .037 |
|  | | CBE | Control | 1.856500 | .854025 | .058 |
|  | |  | CB | 2.093750^*^ | .854025 | .037 |
|  | | Control | CB | -.183500 | 1.150882 | .877 |
|  | |  | CBE | -3.856250^*^ | 1.150882 | .009 |
|  | | CB | Control | .183500 | 1.150882 | .877 |
| pH – 8.5 | |  | CBE | -3.672750^*^ | 1.150882 | .011 |
|  | | CBE | Control | 3.856250^**^ | 1.150882 | .009 |
|  | |  | CB | 3.672750^*^ | 1.150882 | .011 |
